# Vaccination in Children With Autoimmune Disorders and Treated With Various Immunosuppressive Regimens: A Comprehensive Review and Practical Guide

**DOI:** 10.3389/fimmu.2021.711637

**Published:** 2021-08-02

**Authors:** Geraldine Blanchard-Rohner

**Affiliations:** ^1^Paediatric Immunology and Vaccinology Unit, Division of General Paediatrics, Department of Paediatrics, Gynaecology and Obstetrics, Geneva University Hospitals and University of Geneva, Geneva, Switzerland; ^2^Centre for Vaccinology and Neonatal Immunology, Department of Paediatrics and Pathology-Immunology, Medical Faculty and University Hospitals of Geneva, Geneva, Switzerland

**Keywords:** auto-immune, vaccination, children, immunosuppressed, immunomodulatory drugs

## Abstract

Children with autoimmune disorders are especially at risk of vaccine-preventable diseases due to their underlying disease and the immunosuppressive treatment often required for a long period. In addition, vaccine coverage remains too low in this vulnerable population. This can be explained by a fear of possible adverse effects of vaccines under immunosuppression, but also a lack of data and clear recommendations, particularly with regard to vaccination with live vaccines. In this review, the latest literature and recommendations on vaccination in immunosuppressed children are discussed in detail, with the aim to provide a set of practical guidelines on vaccination for specialists caring for children suffering from different autoimmune disorders and treated with various immunosuppressive regimens.

## Introduction

Autoimmune disorders include children with systemic autoimmune diseases and those with autoimmune diseases specific to a single organ, such as the digestive tract (inflammatory bowel diseases), eyes (uveitis), skin (psoriasis) or the central nervous system (multiple sclerosis). These children are treated with similar immunosuppressive therapy to control the disease, which includes traditional immunomodulatory drugs, such as glucocorticoids (GCs), disease-modifying antirheumatic drugs (DMARDs), and biologics. Currently, DMARDs are separated into conventional synthetic (csDMARDs), biological (bDMARDs), and targeted synthetic (tsDMARDs) (1) as shown in **Table 1**.

**Table 1:** List of immunosuppressive agents and current recommendations concerning vaccination [adapted from (2, 3)].

Cellular target	Medication	Administrationroute	Half-life	Low/highdose	Non-live vaccines	Live-attenuated vaccines
**GC-Rec.**	**Gluco-corticoids** **(prednisone)**	Diverse	2–4 h (prednisolone)	Low: <14 days or <0.2–0.5 mg/kg/day or <10 mg/day or substitutive treatment or non-systemic (2, 4–6) High: ≥14 days or ≥2 mg/kg/day (7) or ≥10 mg/kg/day or ≥0.2–0.5 mg/kg/day (2, 4–6) or intravenous pulse methylprednisolone	Anytime but best between 2 and 4 weeks before treatment	- High: Contra-indicated Best 4 weeks before treatment or minimum 1–2 months after treatment (2, 4) - Low: Can be considered for booster immunization with MMR/VZV
***Conventional synthetic DMARDs (csDMARDS)***						
***Inhibitors of DNA/RNA synthesis through the inhibition of the synthesis of one of the nitrogen bases***						
**Pyrimidine synthesis**	**Methotrexate**	Sub- cutanous or oral	3–15 h	Low: <15 mg/m^2^/w High: ≥15 mg/m^2^/w	Anytime, but best between 2 and 4 weeks before treatment	- High: Contra-indicated Best 4 weeks before treatment or 2–3 months after treatment (2, 5, 6) - Low: Can be considered for booster Immunization with MMR/VZV
	**Leflunomide** **(Arava®)**	Intravenous	14 days	Low: ≤0.5 mg/kg/day High: >0.5 mg/kg/day (7)	Anytime, but best between 2 and 4 weeks before treatment - Under treatment, best in the middle of interval	- High: Contra-indicated Best 4 weeks before treatment or 2 years after treatment (long-half-life, if necessary, wash-out option with colestyramin or activated carbon (4) - Low: Can be considered for booster Immunization with MMR/VZV (off-label (2))
**Purine synthesis**	**Azathioprine** **(Imurek®)**	Oral	2 h	Low: ≤3 mg/kg/day (2) High: >1–3 mg/kg/day (7)	Anytime, but best between 2 and 4 weeks before treatment	- High: Contra-indicated Minimum 4 weeks before treatment or 3 months after (2, 4) - Low: Can be considered for booster immunization with MMR, VZV (2)
	**6-mercaptopurine (6MP)**		2 h	Low: ≤1.5 mg/kg/day (4) High: >1.5 mg/kg/day (7)	Anytime, but best between 2 and 4 weeks before treatment	- High: Contra-indicated Minimum 4 weeks before treatment or 3 months after (2, 4) -Low: Can be considered for booster immunization with MMR, VZV (4)
	Mycophenolate-mofetil (CellCept®)	Oral	17 h	Low: ≤1,200 mg/m^2^ High: >1,200 mg/m^2^ (2)	Anytime, but best between 2 and 4 weeks before treatment	High: Contraindicated Minimum 4 weeks before treatment or 1–3 months after (2, 4–6) - Low: Can be considered for booster immunization with MMR, VZV (4)
**Alkylating agents** **(DNA synthesis)**	Cyclophosphamide (Endoxan®)	Oral Intravenous	3–12 h	0.5–2 mg/kg/day in single oral dose or as intravenous pulse every 2–4 weeks	Anytime, but best between 2 and 4 weeks before treatment During treatment, best in the middle of interval	Contraindicated during treatment, minimum 4 weeks before or 1–3 months after (2, 4–6)
***Inhibitors of intracellular signal transduction from the antigen-recognizing TCR through the inhibition of calcineurine pathways***						
**Calcineurin inhibitors**	Ciclosporine (Sandimmun®)	Oral	7–19 h	Low: ≤2.5 mg/kg/day High: >2.5 mg/kg/day	Anytime, but best between 2 and 4 weeks before treatment	High: Contraindicated Minimum 4 weeks before treatment or 1–3 months after (2, 4–6) -Low: Can be considered for booster immunization with MMR, VZV (4)
	Tacrolimus	Oral	23–46 h	High interpatient and intrapatient variability, monitoring of levels necessary	Anytime, but best between 2 and 4 weeks before treatment	High: Contra-indicated Minimum 1–4 weeks before treatment or 1–3 months after (2, 4–6) -Low: According to studies in liver transplant recipients, dosage of <0.3 mg/kg/day tacrolimus (blood level <8 ng/ml) can consider live vaccines (8, 9)
**Inhibitors of mammalian target of rapamycin (mTOR)**	Sirolimus Everolimus	Oral	62 h 30 h	High interpatient and intrapatient variability, monitoring of levels necessary	Anytime, but best between 2 and 4 weeks before treatment	High: Contraindicated Minimum 4 weeks before treatment or 1–3 months after and verify CD4 and CD19 before (5, 10)
***Other molecules considered as csDMARDs with little or no immunosuppressive effect***						
**PGLs synthesis** **(analogue of 5-ASA)**	Sulfasalazine (Azufidine®)	Oral	6–8 h (11)	Low: ≤40 mg/kg/day High: >4 0 mg/kg/day or 2 g/day (2)	Anytime best between 2 and 4 weeks before treatment	OK, no pause of treatment required; however, oral typhus vaccine should be administered at least 24 h after administration of sulfasalazine (2)
	Mesalazine (Pentasa®, Asacol®, Claversal®, Salofalk®)	Oral	5 h	Standard dose	Anytime best between 2 and 4 weeks before treatment	No restriction, no interruption
**Antimalarials**	Hydroxychloroqine	Oral	40 days	6.5 mmg/kg/day (max 400 mmg/day)	Anytime best 2 and 4 weeks before treatment	No restriction, no interruption
**Others**	Colchicine	Oral	13 h	0.5–2 mg/day	Anytime best 2–4 weeks before treatment	No restriction, no interruption
	Thalidomide	Oral	7 h	2.5–4 mg/kg/day	Anytime best 2–4 weeks before treatment	No restriction, no interruption
***Biological DMARDs (bDMARDs)***						
**Anti-TNF-**	Adalimumab (Humira®) mAb to TNFα	Sub-cutaneous	10–20 days	15–30 kg: 20 mg every 2 weeks >30 kg: : 40 mg every 2nd week	Anytime best 2–4 weeks before treatment, or in the middle of the treatment interval	- Best 4 weeks before starting treatment or 3 months after last dose - for newborns exposed during pregnancy, wait a minimum of 5 months after last dose during pregnancy (2) - Breastfeeding: OK (2)
	Infliximab (Remicade®) mAb to TNFα	Intravenous	12 days	6–10 mg/kg intravenous on weeks 0, 2 and 6, then every 4–8 weeks	Anytime best 2–4 weeks before treatment, or in the middle of the treatment interval	- Best 4 weeks before starting treatment or 3 months after last dose - for newborns exposed during pregnancy, wait a minimum of 6 months after last dose during pregnancy (2)
	Golimubab (Simponi®) mAb to TNFα	Sub-cutaneous	14 days	50 mg 1×/month	Anytime best 2–4 weeks before treatment, or in the middle of the treatment interval	- Best 4 weeks before starting treatment or 3 months after last dose - for newborns exposed during pregnancy, wait a minimum of 6 months after last dose during pregnancy (2)
	Certolizumab pegol (Cimzia®) Pegylated mAb to TNFα	Sub-cutaneous	14 days	200–400 mg every 2 weeks	Anytime best between 2 and 4 weeks before treatment During ongoing treatment, administration in the middle of the treatment interval	- Best 4 weeks before starting treatment or 3 months after last dose - for newborns exposed during pregnancy, wait a minimum of 5 months after last dose during pregnancy (2) - Breastfeeding: OK (2)
	Etanercept (Enbrel®, Erelzi®) TNFRII/FcIgG1	Sub-cutaneous	70 h	0.4 mg/kg 2×/weekly Or 0.8 mg/kg/week Subcutaneous: maximum 50 mg/week	Anytime best 2–4 weeks before treatment	- Best 4 weeks before starting treatment or 1–2 months after last dose (2, 10) - for newborns exposed during pregnancy, wait a minimum of 4 months after last dose during pregnancy (2) - Breastfeeding: OK (2)
**Anti-IL-1**	Anakinra (Kineret®) IL1-Rec1	Sub-cutaneous	4–6 hours	1 mg/kg subcutaneous daily (max 100 mg)	Anytime best between 2 and 4 weeks before treatment	- Best 4 weeks before starting treatment or 2–4 weeks after last dose (2, 10)
	Rilonacept (Arcalyst®) IL1R/IL1AcP/IgG1	Sub-cutaneous	1 week	2.2 mg/kg 1×/week (initiate with double dose)	Anytime best between 2 and 4 weeks before treatment	- Best 4 weeks before starting treatment or probably 1 month after last dose
	Canakinumab (Ilaris®) mAb to IL1β-Rec	Sub-cutaneous	30 days	Low: 2 mg/kg	Anytime best between 2 and 4 weeks before treatment During treatment: in the middle of the interval	- Best 4 weeks before starting treatment or 7 months after last dose (10) - During treatment: minimum of 3 months after the last and before the next injection (2) - Newborns exposed *in utero* should wait 16 weeks after delivery for live-attenuated vaccine (2)
**Anti-IL-6R**	Tocilizumab (RoActemra®) mAb to IL6R	Intravenous	6–23 days	Poly JIA >2 years, <30 kg, 10 mg/kg, every 4 weeks >2 years, >30 kg, 8 mg/kg every 4 weeks SJIA >2 years, <30 kg 12 mg/kg every 2 weeks >2 years, >30 kg, 8 mg/kg every 2 weeks	Anytime best 2–4 weeks before treatment, or in the middle of the interval of doses	- Best 4 weeks before starting treatment or 2–3 months after stopping treatment (1, 2, 10)
**CD80/CD86- Rec.**	Abatacept (Orencia®) CTLA4-Ig	Iv	13 days	Standard dose: 10 mg/kg weeks 0, 2, 4 then q4 week	Anytime Best 2–4 weeks before treatment, or in the middle of the interval of doses	- Best 4 weeks before starting treatment or 3 months after stopping treatment (2, 10) - for newborns exposed during pregnancy: wait 14 weeks before live vaccine (2)
**B-cell inhibitor**	Rituximab (MabThera®) mAb to CD20	Intravenous	20.8 days	375–500 mg/m^2^ intravenous every 2 weeks × 2 dose	- 4 weeks (2 weeks) before treatment start -6 months after last dose (2)	- Minimum of 6 weeks before starting treatment - 12 months after last dose and after checking restoration of B cells (2) -for newborns exposed during pregnancy: wait for normalization of B cells (2)
	Ocrelizumab (Ocrevus® ) mAb to CD20	Intravenous	28 days	300 mg intravenous every 2 weeks × 2 doses, then 600 mg every 6 months	6 weeks (2 weeks) before treatment start -6 months after last dose (2)	- Minimum of 6 weeks before starting treatment (2) - 18 months after last dose and after checking restoration of B cells (2) -for newborns exposed during pregnancy: wait for normalization of B cells (2)
	Belimumab (Benlysta®) mAb to B-cell activating factor (BLys)	Intravenous	12.5–19.4 days	10 mg/kg every 2 weeks × 3 then every 4 weeks	Anytime best 2–4 weeks before treatment or in the middle of the interval of doses	- Best 4 weeks before starting treatment - 3 months after stopping treatment (2)
**Anti-C5**	Eculizumab (Soliris®)	Intravenous	12 days	Various doses, depending on indications	Vaccination against *N. meningitidis* at least 2 weeks before starting treatment (10)	- Best 4 weeks before starting treatment - 32 months after stopping treatment (10)
**Integrin α_4_β_7_ (LPAM-1, lymphocyte Peyer patch adhesion molecule 1)**	Vedolizumab (Entyvio®)	Intravenous	25 days	Between 100 and 300 mg intravenous at weeks 0, 2, and 6, then every 8 weeks	Anytime best 2–4 weeks before treatment (2)	Anytime, except for live vaccines, should be given at least 3 months after stopping treatment (2, 10)
***Targeted synthetic DMARDs***						
**JAK inhibitor**	Baricitinib (Olumiant®)	Oral	12.5 h	4 or 8 mg daily	Anytime best 2 weeks before or 4 weeks after treatment	- Best 4 weeks before starting treatment - 1 month after stopping treatment
	Tofacitinib (Xeljanz®)	Oral	3 h (11)	Low: ≤5–10 mg/day High: >10 mg/kg/day	Anytime best 2–4 weeks before treatment	- Best 4 weeks before starting treatment - 2 months after stopping treatment (2) - Low dose: depending on the risk, vaccination with MMR, VZV or MMR-VZV might be possible (2)
**Phosphodiesterase 4 inhibitors**	Apremilast (Otezla®)	Oral	8.9–9.7 h (11)		Anytime Best 2 weeks before or 4 weeks after stopping treatment	- Best 4 weeks before starting treatment or 2 weeks after stopping treatment (10)

Children with autoimmune disorders are at an increased risk of infection due to their underlying disease and to their immunosuppressive treatment. A study assessing the risk of infection every 2 months for one year in juvenile idiopathic arthritis-children treated with bDMARDs reported that 57% (n = 175) of patients developed an infection. Upper respiratory tract infections were among the most frequent infections and mostly treated in ambulatory care (12).

Several studies have reported similar rates of serious infection in children with JIA receiving bDMARDs or csDMARDs (12, 13). However, other studies have reported that the highest rates of infection were in children treated with bDMARDs, especially anti-TNFα (such as etanercept and infliximab) (12, 14–16), or a combined treatment of csDMARDs (such as MTX) and bDMARDs (17). Upper respiratory tract infections (including severe influenza) were among the most frequent infections, together with complicated varicella (12, 14–19).

As most patients received a combined treatment rather than a single molecule, it is particularly challenging to design a clinical study to assess the effect of various immunosuppressive regimens on the risk of infection and to understand the biology behind the infectious risk.

Infectious diseases for which a vaccine is available for children include influenza virus, *S. pneumoniae*, *H. influenzae*, meningococcus, polioviruses, VZV, measles, mumps, rubella, HPV, hepatitis A (HAV) and B virus (HBV), tick-borne encephalitis, and travel vaccines (such as the yellow fever vaccine, rabies vaccine, and typhoid vaccine).

In this review, the challenges in vaccinating children with autoimmune and autoinflammatory disorders is discussed and how the immune response develops following vaccination and how it is affected by various immunosuppressive drugs is explained. Then, the review focuses on live vaccines, discusses the existing data, the controversies and existing recommendations, and does the same for non-live vaccine. At the end, a practical approach for vaccinating children with immune-mediated disorders is proposed. In this review, vaccination in function of the immunosuppressive regimen rather that in function of the various autoimmune and autoinflammatory disorders is discussed. Further, it should be noted that the majority of studies that we have in this field come from studies mainly done in children with JIA, SLE, and autoinflammatory disorders, and few studies in children with IBD, while there are almost no studies for other autoimmune diseases.

## Challenges in the Vaccination of Children With Autoimmune Disorders

One of the major achievements in medicine is the development of vaccines, which allow protection against many potentially fatal infectious diseases, thus decreasing mortality worldwide. However, recent outbreaks of vaccine-preventable diseases, such as measles, show that reaching a sufficient vaccine coverage of the international population remains a challenge (20, 21).

Completion of vaccination series is even more important in children with autoimmune disorders. First, they are more susceptible to infections because of the underlying conditions that affect their immune system and influence their natural defense mechanisms against various infectious agents. Furthermore, these children often require a rapid start of immunosuppressive treatment after diagnosis, usually lasting for many months/years until the treatment can be reduced or interrupted, which renders vaccination challenging in this population. Indeed, it is expected that most immunosuppressive drugs will affect the immune capacity of the child to a different degree depending on the agent, thereby reducing their capacity to respond to many vaccines. In addition, only non-live vaccines are recommended during immunosuppressive treatment and the use of live-attenuated vaccines should be carefully assessed on a case-by-case basis.

In the specific population of children with immune-mediated disorders treated with various immunosuppressive agents, the indication for each vaccine can be complicated, and it becomes very challenging for the specialists who care for these children to decide upon the best vaccination scheme for their patients. Moreover, several concerns, misconceptions, and unanswered questions have led to decreased vaccination rates in children with chronic autoimmune diseases (22, 23). For example, in Ljublijana, Slovenia, only 65% of 18-year-old young adults with rheumatic diseases were up to date with their vaccines, with the most frequently omitted being hepatitis B (HBV) and a second dose of measles–mumps–rubella (MMR) (24). In addition, only 10% had received the seasonal influenza vaccine and 4% the pneumococcal 13-valent conjugate vaccine (PCV13) (24). Similarly, 40% of children with JIA in Canada have an incomplete vaccination record for their age (23).

The reasons described for these decreased rates were that medical specialists caring for patients with autoimmune diseases did not feel responsible for monitoring the vaccination schedules of their patients (25). Additionally, parents—and even specialists—remained uncertain about the safety of some vaccines in the context of children with autoimmune diseases and under immunosuppressive treatment (23). Safety aspects in terms of the potential interferences of vaccination on the underlying disease, as well as the question of whether vaccination under immunosuppressive treatment is sufficiently immunogenic/protective, are repeatedly subjects of discussion and debate (7, 26, 27). Specialists very much worry about the potential danger of administering a live-attenuated vaccine in a child with an autoimmune disease, even under low-immunosuppression, because of the risk of causing vaccine virus associated infections. In addition, current vaccine recommendations for pediatric populations with immune-mediated disorders are often based on small sample sizes with low levels of evidence, especially for the use of live-attenuated vaccines (7, 26, 27).

## Immune Responses To Vaccination

**Figure 1:**
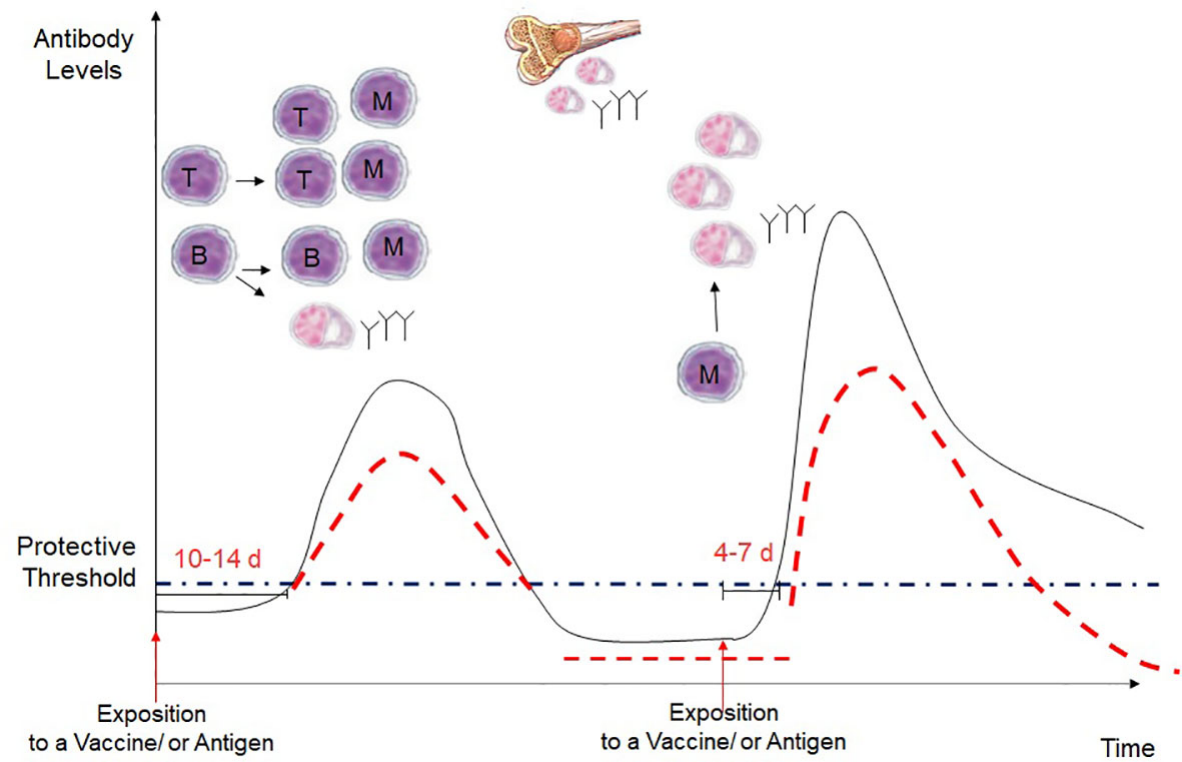
Kinetics of the antibody response in a primary and secondary immune response. Following a first exposure with an antigen, there is an interval of 10-14 days before the production of IgG antibodies, which occurs after the interaction of naive B and T cells, which become effector cells and develop into gentianée-specific memory B cells, short-lived plasma cells and long-lived plsame cells. Then, long-lived plasma cells in migrate in the bone marrow and continue to secrete IgG antibodies. However, with the time the antibodies decrease often under the protective levels. During a secondary exposure with the vaccine or the antigen, memory B and T cells are rapidly reactivated, with a more rapid and higher IgG increase that usually last longer. However, in immunosuppressed children, the peak of the antibody response is expected to be lower, and the antibodies are expected to decrease more rapidly after vaccination.

During a first encounter with an antigen, only a small number of naïve B cells and T cells are able to recognize a given antigen. After around 2 weeks, clones of T and B cells are selected, expand, and give rise to a small pool of memory B and T cells, which are often too small and last for a too short period to offer protection against a given pathogen. Following subsequent encounters with the given antigen, memory B and T cells proliferate and expand. These cells respond more rapidly and more strongly following a smaller amount of antigen. This explains the principle of vaccines, which allows for producing a pool of memory B and T cells able to respond rapidly to a given antigen after infection and also giving rise to long-lived plasma cells that persist in the bone marrow (28) (**Figure 1**).

To better understand the effect of each immunosuppressive drug on the immune response to vaccination, we should look at what happens specifically at the cellular level. Following a first exposure with the antigen, naïve T cells in lymph nodes recognize a peptide antigen presented on the surface of antigen-presenting cells (APCs) in the MHC molecule *via* the binding of their T-cell receptor (TCR) and co-stimulatory signals given by the CD80 and CD86 on the surface of DCs and CD28 on T cells. The extent and quality of antigen-presenting cell activations condition the T-cell responses. T-cell responses are often dependent on the inflammatory milieu (innate immune responses) created at the time of vaccination and these could be impacted by the various immunosuppressive drugs. This activates various signal transduction pathways in T cells, which activate the transcription of various factors that induce the expression of several molecules and, most importantly, IL-2, which binds to the CD25 receptor on the surface of activated T cells and induces its survival and proliferation. After 4–5 days of division, the activated T cells differentiate into effector and memory T cells (29, 30).

**Figure 2:**
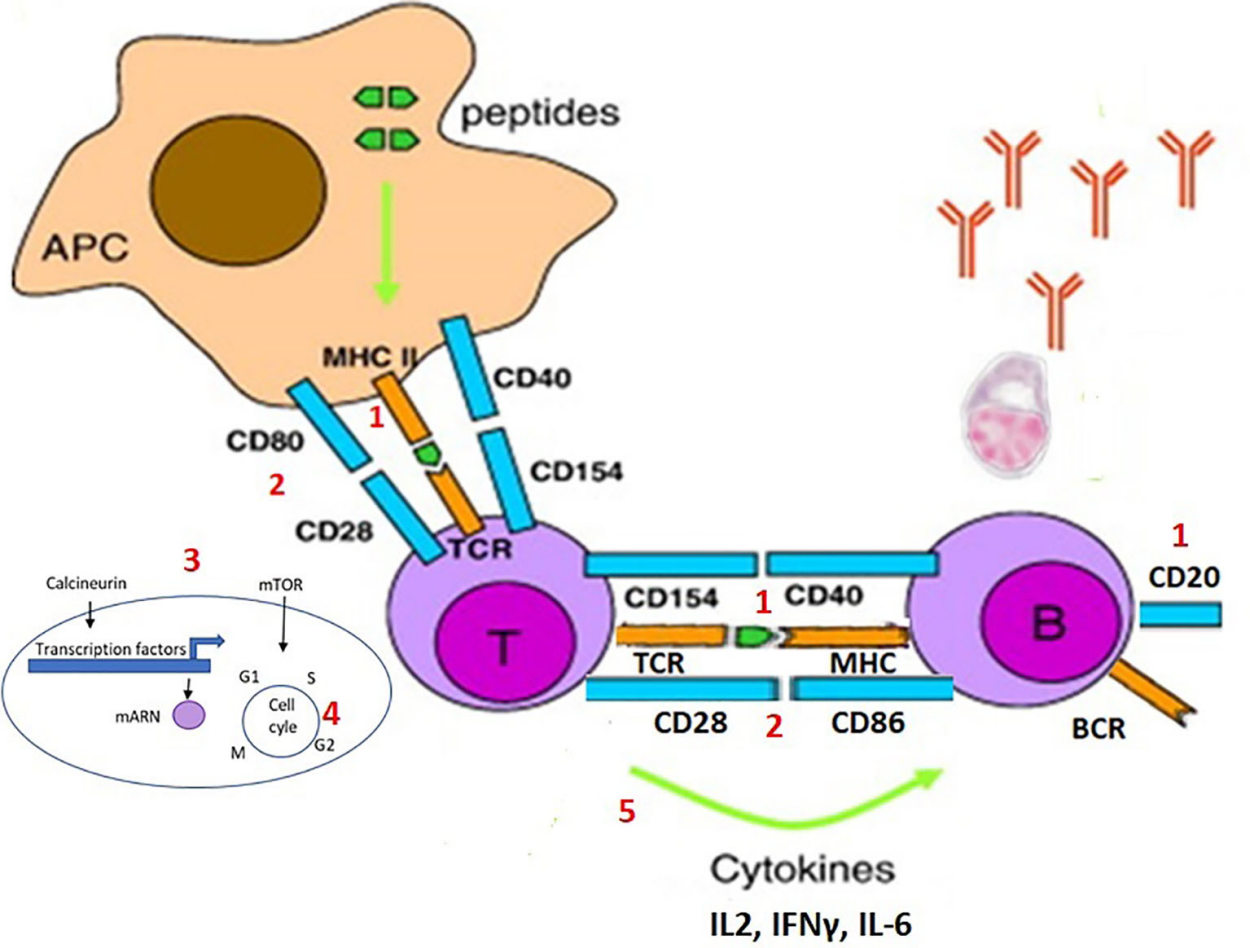
Steps of the activation and proliferation of T cells and B cells during an immune response, and possible targets of the immunosuppresive drugs. During an immune response, naïve T cells recognize a peptide antigen presented on the surface of antigen-presenting cells (APCs) in the MHC molecule via the binding of their T-cell receptor (TCR) and co-stimulatory signals given by the CD80 and CD86 on the surface of DCs and CD28 on T cells. This activates various signal transduction pathways in T cells, which activate the transcription of various factors that induce the expression of several molecules, such as IL-2. Naïve B cells that have bound antigen to their surface Ig receptors require co-stimulatory signals from T cells that are specific for the same antigen. This allows initiating a germinal center reaction with proliferation and mutation of the antibody genes and then differentiate into antibody-producing plasma cells and memory B cells. Each of the steps of the immune response can be the target of an immunosuppressive drug: 1) the depletion of the specific or cognate T and/or B cells (e.g. anti-CD20); 2) interference with the co-stimulatory signals (e.g. CTLA4-analog); 3) blockade of the intracellular signal (e.g. calcineurin inhibitor or mTOR inhibitor); 4) inhibition of DNA synthesis and cell proliferation (e.g. purine analog, or alkylating agents); 5) modulation of the effector T or B cell responses (various anticytokine monoclonal antibodies), included blocking inflammation reducing antigen presentation (anti-IL6, TNF, JAK, etc.).

Naïve B cells that have bound antigen to their surface Ig receptors require co-stimulatory signals from CD4 helper T cells that are specific for the same antigen. This allows initiating a germinal center reaction in secondary lymph nodes and proliferating and mutating their antibody genes through somatic hypermutation to achieve higher affinity and then differentiate into antibody-producing plasma cells and memory B cells (**Figure 2**).

## General Principles of the Effect of Immunosuppressive Drugs on the Immune Response to Vaccination

Each of the steps of the immune response can be the target of an immunosuppressive drug (**Figure 2**) (29): 1) the depletion of the specific or cognate T and/or B cells (*e.g.* anti-CD20); 2) interference with the co-stimulatory signals (*e.g.* CTLA4-analog); 3) blockade of the intracellular signal of the antigen-recognizing TCR (*e.g.* calcineurin inhibitor or mTOR inhibitor); 4) inhibition of DNA synthesis and cell proliferation (*e.g.* purine analog, or alkylating agents); 5) modulation of the effector T or B cell responses (various anticytokine monoclonal antibodies), included blocking inflammation reducing antigen presentation (anti-IL6, TNF, JAK, *etc.*).

Overall, the result is that the various immunosuppressors block the clonal expansion of specific T and B cells. In general, the primary immune response to a novel antigen is typically more severely affected than a secondary immune response, as the threshold for activation of memory B and T cells is lower. However, some drugs may affect directly on T/B cell activation/trafficking. In addition, the clonal expansion of the memory cells is reduced, and the antibodies are produced in a lower amount and of reduced quality and therefore resulting in a shorter duration of protection.

Classical vaccines can be subdivided into two groups including the live-attenuated vaccines and the inactivated-subunit-killed vaccines (commonly named “non-live” vaccines). These groups differ in the way they stimulate the immune system. Inactivated vaccines are used against bacteria and viruses that cannot be attenuated. Their advantage is that the product is chemically defined, stable, safe and contains only B cell and T-cell specific epitopes. They can be administered without any risk in any patient, including those who are immunosuppressed. However, they require frequent booster immunizations.

Live-attenuated viral vaccines can be created with less virulent (attenuated) mutants of the wild-type virus or with viruses from other species that share antigenic determinants. Virus can be attenuated *via* passage through a foreign host, such as embryonated eggs or tissue culture, where they acquire mutations to infect the new host. The new virus population will be significantly different from the initial population and will not grow well in the original host. The disadvantages of these vaccines are that they require refrigeration and they cannot usually be administered to immunocompromised patients because of the risk of disease caused by the vaccine strains.

There is also the possibility to create vaccines with viruses that lack virulence properties using genetic engineering. Molecular techniques are now being used to develop new vaccines. By genetic engineering, new live-attenuated vaccines can be generated by the induction of mutations to delete or inactivate genes encoding virulence factors. These new techniques appear to be more reliable than random attenuation of the virus *via* passage through tissue culture. Hybrid virus vaccines can be formed when genes from infectious agents that cannot be easily attenuated are inserted into safe viruses. A defective, infectious, single-cycle (DISC) virus vaccine is formed by a virus with a deletion of an essential gene that is grown in a tissue culture cell that expresses the defective gene. In DNA vaccines, the genes coding for a protein that expresses an important B- and T-cell-specific viral or bacterial epitopes are inserted into a plasmid vector, thus permitting the protein to be expressed in eukaryotic cells. Plasmid DNA is injected into muscle or skin, and then taken up by DCs where the cDNA is transcribed and the immunogenic protein expressed, permitting the induction of a cell-mediated and humoral immune response. Attenuated viruses or bacteria, such as *Escherichia coli*, may be used as vectors containing the plasmid. Reverse vaccinology, which utilizes genomic sequence data, is a new approach for the development of vaccines. These new technologies are exploding in the context of the current severe acute respiratory syndrome coronavirus 2 (SARS-CoV-2) pandemic and it is anticipated that they will also influence the development of new vaccines for different populations such as the “too young, too old, or too immunocompromised”. Hopefully, these new vaccines and also the development of new adjuvant will help in developing vaccines that are more adapted to immunosuppressed patients.

Revaccination with non-live vaccine is therefore expected to induce a reduced immune response in immunosuppressed compared to healthy children. In contrast, during a primary vaccination with a novel antigen, additional doses may be necessary to reach the protective antibody threshold. For this reason, when possible, it is very important to verify the antibody titer post-vaccination to decide on whether additional doses must be given. In addition, following immunization with live-attenuated vaccines, there is the risk that the inhibition of the clonal expansion of T and B cells may lead to the inability to clear the replicating attenuated vaccine-strain virus, leading to the possibility of severe vaccine-associated disease and adverse events.

The introduction of bDMARDs in 1995 has revolutionized the treatment of autoimmune diseases, as these new treatments target specific pathways that are involved in immune function, inflammation and autoimmunity, rather than broadly suppress cell activation/proliferation as csDMARDs. The advantage is that bDMARDs are more targeted and effective in treating autoimmune diseases. Of note, there is a continuous introduction of new monoclonal antibodies, but only those currently used in children are listed in **Table 1**, classified according to their mode of action. The current recommendations of vaccination with non-live and live-attenuated vaccines are summarized for each type of immunosuppressive drug. There will be a need to revise and adapt vaccination guidelines regularly due to constant advances in this field.

## Vaccination With Live-Attenuated Vaccines

Vaccination schedules for MMR and VZV vaccines differ among countries. While the first MMR vaccine dose is given around 9–15 months of age in all countries, the timing of the second dose varies greatly. It is recommended before the age of 2 years in Switzerland and Australia, or between 4 and 6 years in countries such as France, Spain, the United Kingdom, USA and Canada, or even as late as at 9 years old in Hungary, The Netherlands, Estonia, Norway, Poland and the Slovak Republic (17, 31–33). Most European countries do not vaccinate against varicella, while VZV vaccine is part of the routine vaccination schedule in Australia, Canada, and the USA. Hence, depending on the age at onset of the autoimmune disease, the child might not be immune against measles and varicella at the time of diagnosis. In healthy children, VZV vaccine is 82% effective at preventing any form of varicella after one dose and 98% after 2 doses, but 100% effective at preventing severe varicella after one dose (34). MMR vaccine is very safe and effective in healthy people. One dose of the vaccine in 93% effective, while two doses are 97% effective at preventing measles (34).

The risk of measles and varicella infections in immunocompromised children is even more important at the current time of increasing vaccine hesitancy and measle outbreaks worldwide. Therefore, assuring a protective immunity against measles and varicella in immunocompromised children can be very challenging. Once the immunosuppressive treatment has been introduced, it is no longer possible to vaccinate against these diseases, as only live-attenuated vaccines are available. Furthermore, vaccinating children during the acute phase of disease with a live-attenuated vaccine is often difficult as a time interval of minimum 4 weeks is necessary between vaccination and the beginning of the immunosuppression, and even more if two doses are needed.

Previous studies that have assessed the safety and immunogenicity of live-attenuated vaccines in children on immunosuppressive treatment are summarized in **Table 2**. There are almost no data on primary vaccination with MMR in immunosuppressed children as the first dose of this vaccine is typically given before the onset of most autoimmune disorders.

**Table 2:** Summary of previous studies on live-attenuated vaccines in immunocompromised children.

Author/year/country	Vaccine	Study design	Disease	No. of patients and treatment/dosage	Safety	Immunogenicity
MMR						
Heijstek 2007 Netherlands (35)	MMR	Retrospective Questionnaire to patients	JIA	- 207 JIA patients - 49 treated with MTX - 158 without MTX MTX: median dose 11 mg/m^2^/week	-No increase in disease activity -No overt measles infection	NA
*Borte 2009 Germany (36)	MMR booster	Prospective	JIA	-15 JIA patients --5 MTX alone --5 MTX + etanercept --5 patients on MTX 4 years post-MMR --22 healthy controls MTX 10 mg/m^2^/week Etanercept 0.4 mg/kg 2×/weekly	-No increase in disease activity -No overt MMR infections in 10 patients vaccinated under MTX alone or MTX + etanercept	-No impact of both MTX alone or combined with etanercept on antibody and T-cell responses. -Trend towards lower antibody titers in JIA-patients treated with MTX compared to healthy children in the long-term, but higher virus-specific IFNgamma-producing T cells
Miyamoto 2011 Brazil (37)	MMR	Retrospective	pSLE	30 SLE on various treatments (25 HCQ, 19 oral GCs, 14 AZA, 9 intravenous GC, 2 CFM pulse, 2 CY, 2 MTX, 1 MMF)		At 7–16 years post-immunisation, good maintenance of antibodies for measles
Heljstek 2012 Netherlands (38)	MMR booster	Retrospective	JIA	400 JIA (246 nonsteroidal anti-inflammatory drugs, 93 MTX, 28 oral GC, 24 DMARD, 8 anti-TNF)		Long-term Ab levels lower for rubella and mumps up to 10 years of post-vaccination, but normal for measles
*Heijstek 2013 Netherlands (39)	MMR booster	Randomized controlled trial	JIA	- 137 JIA patients - 63 were vaccinated (29 patients on low-dose MTX, 9 patients on anti-TNF or anti-IL1, briefly interrupted at time of immunization) -68 patients not vaccinated	No increase in disease activity or disease flares in the 12 months following vaccination	-Higher antibody titres in patients vaccinated -Seroprotection rates between 97 and 100%, even 12 months post-vaccination. -No patients developed overt vaccine strain viral infection
Uziel 2020 (40) 10 countries	Booster of MMR/MMRV	Retrospective, multicenter	234 patients with rheumatic disease (90% JIA)	124 MTX 71 MTX + biologics 39 biologics only Biologics included anti-TNFa (41), anti-IL1 (42) and anti-IL-6 (7).	MMR/VZV was safe 13 mild adverse events (skin reaction, local pain, mild fever, flu-like symptoms)	NA
Maritsi 2019 (42)	two doses of MMR	Prospective study on long-term persistence of Abs after MMR	41 with enthesitis JIA	On anti-TNF		Measles and rubella Ab loss is accelerated, but seroprotection is retained
VZV						
Pileggi 2010 Brazil (43)	Primary dose of VZV	Prospective	Rheumatic disease	-25 patients (17 JIA, 4 juvenile dermatomyositis, 4 other rheumatic diseases) -5 MTX + DMARDs -13 MTX + GCs GC 0.1–0.7 mg/kg/d MTX 12–25 mg/m2/w CY 3–3.5 mg/kg/d Leflunomide 10 mg/d Penicillamine 13 mg/kg/d	-No increase in disease activity -No over VZV infection -three patients with mild self-limited VZV-like rash	-Slight decrease in seroresponse in patients compared to controls -two of eight patients exposed to VZV developed chickenpox, 1 was on anti-TNFalfa -At one year, 80% patients maintained VZV Abs
Lu 2010 North America (44)	VZV	Case series	six IBD	6 6-MP 2 anti-TNF	No serious adverse events after primary/booster VZV, despite anti-TNFalfa	Seroprotection in 5/6 patients
*Barbosa 2012 Brazil (45)	VZV booster	Randomized controlled trial	54 SLE	28 SLE vaccinated -27 HCQ -18 GCs low-dose -9AZA -2MTX 26 SLE non-vaccinated -22 HCQ -18 GC low-dose -12 AZA -2 CY	No increase in disease flare among vaccinated patients	-Similar antibody response at short term -Over 35.6 months after vaccination, four cases of HZ in the unvaccinated group compared to none in the vaccinated group
*Toplak 2015 Slovenia (46)	Primary dose of VZV	Prospective	JIA	six patients on biologics (three on etanercept, two on tocilizumab, 1 on infliximab) four patients received first dose of VZV	Vaccine was safe: no severe adverse events and no varicella infection Stable disease activity	-5/6 patients produced protective Abs after 2nd dose -1/6 did not and had a mild varicella infection 4 months after the second vaccination. -Production of Abs higher in children on tocilizumab than in those on etanercept -In the long term, Abs declined in children on tocilizumab
*Groot 2017 Brazil /Netherlands (47)	VZV booster	Prospective	Various rheumatic diseases	49 patients (39 JIA, 5 juvenile dermatomyositis,, 5 Juvenile systemic sclerosis) and 18 healthy controls All patients were on MTX, 16 on GC and 3 on biologics (adalimumab, etanercept and abatacept)	Vaccination was safe No disease flare	-Good Ab response and cellular response. -No effect of various immunosuppressive treatments. -Second dose (n = 21) increased VZV Ab
*Speth 2018 Germany (48)	VZV (booster and primary dose)	Prospective Based on a pre-vaccination checklist	Rheumatic disease	23 patients with RD 9 on biologics (4 anti-TNF, 2 anti-IL-1, 2 anti-IL-6, 1 abatacept) (9/23 had already received one dose of VZV) 9 on low immunosuppressive treatment 14 on high IS	Mild adverse events, no severe adverse events, no rash or vaccine-induced VZV, no disease flare	Good Abs response, even for the low and high immunosuppressive treatments 21/23 responded after first dose. 2/23 patients failed to respond.
Jeyaratnam 2018 (49)	Various live-attenuated vaccine	Retrospective multicenter survey	Patients on IL-1 and IL-6 blockers	17 patients with auto-inflammatory disorder sJIA, 5 CAPS, 4 MKD, 1 FMF 14 on anti-IL-1, 3 on anti-IL-6 7 received MMR boost 1 received first dose of MMR/VZV while on canakinumab, which was stopped at time of vaccination 1 received first dose of VZV while on tocilizumab 4 received first Yellow fever 1 received oral polio while on tocilizumab	two patients had severe adverse events: varicella zoster infection after VZV booster (in a child on anakinra, low-dose GCs and several DMARDs), and a pneumonia after MMR booster (in a child on canakinumab, low-dose GCs and MTX). seven patients had a mild disease flare eight patients had no disease flare	NA

* = prospective studies; NA = not available.

### Evidence for Safety and Immunogenicity of Live-Attenuated Vaccines

#### Measles, Mumps, Rubella

In a prospective, nested, case–control study, the immune response following a booster dose of MMR was comparable in both healthy controls and 15 children with JIA treated with low-dose MTX + or anti-TNFα (etanercept) (36). A Dutch randomized, multicenter, open-label clinical equivalence trial assessed the effect of a MMR booster dose in 137 JIA patients aged between 4 and 9 years (60, MTX; 15, bDMARDs) in which patients were randomly assigned to receive MMR booster or placebo. Among patients taking bDMARDs, treatments were interrupted five times their half-lives prior to vaccination. The authors observed a good immunogenicity of the booster dose of MMR in JIA patients and no increase in disease flares in the year following vaccination (39). A retrospective, single-center Dutch study compared the long-term persistence of antibody to MMR, diphtheria, and tetanus toxoids in 400 JIA patients compared to 2,176 healthy controls. They reported lower levels of antigen-specific antibodies in JIA patients for all antigens, except measles, although seroprotection rates were similar in JIA patients and controls. Furthermore, the use of MTX and GCs had no effect on antibody persistence (38). Other studies have reported that revaccination with MMR in patients treated with various immunosuppressive treatments was safe and immunogenic, although the antibody response was lower in the short and longer term (35, 37, 40, 42) (**Table 2**).

#### Varicella (Chickenpox) Vaccine

In a prospective controlled study, 25 children with various rheumatic diseases (seventeen JIA, four juvenile dermatomyositis, three juvenile scleroderma, one vasculitis), treated with MTX alone or with prednisone (maximum 10 mg/day) or other csDMARDs, received a single primary dose of VZV vaccine. Three patients with JIA presented a mild self-limited varicella-like rash in the first 2 weeks post-vaccination, without any other symptoms, and the rash spontaneously resolved after 5 to 7 days. More importantly, the number of active joints in JIA patients significantly decreased at month 3 after vaccination (43). In another prospective controlled study, 54 children with SLE treated with various csDMARDs and immune for varicella were randomly assigned to receive a single booster dose of VZV vaccine or placebo. There was no difference in the rates of adverse events or frequency of SLE flares between the vaccinated and non-vaccinated children (45). A case series reported the administration of a first dose of VZV vaccine in 4/6 children with JIA treated with bDMARDs. They reported that the vaccine was safe, but not efficacious in all children as one patient did not respond and presented a mild varicella infection 4 months later. Although it was a very small sample size, it appears that patients treated with anti-TNFα (etanercept) responded less well (46). Another case–control study assessed the immune response to a booster dose of VZV vaccine in 49 children with diverse rheumatic diseases (three of whom were treated with bDMARDs) compared to 18 healthy controls. They reported good safety data and similar humoral responses in patients compared to healthy controls (47). Similarly, another prospective study assessed the immune response to primary and booster doses of VZV vaccine in children on immunosuppressive treatments, nine of whom were on bDMARDs. They used a pre-vaccination checklist with basic laboratory tests: white blood cell count ≥3,000/mm^3^, lymphocytes ≥1,200/mm^3^, serum IgG ≥500 mg/dl, IgM ≥20 mg/dl, tetanus toxoid antibody ≥0.1 IU/ml. In the case of high immunosuppression, additional specifications included a CD4+ lymphocyte count ≥200/mm^3^ and a positive T-cell function (*via* the positive control of a standard tuberculosis interferon-gamma-release-assay indicating mitogen-induced T-cell proliferation). Patients who met the criteria of the pre-vaccination checklist received the first and/or second VZV vaccination, with good safety and immunogenicity results (48).

A retrospective multicenter survey in which physicians treating children with autoinflammatory diseases on anti-IL-1 and anti-IL-6 were contacted and asked to report safety data concerning the vaccination with live-attenuated vaccines. Good safety data were reported concerning 17 children (seven with sJIA and ten with periodic fever syndromes), apart from two serious adverse effects: a VZV infection after a VZV booster in a child on anti-IL-1 (anakinra), low GCs and several csDMARDs and a pneumonia after a MMR booster in a child on anti-IL-1 (canakinumab), low GCs and MTX (49). Finally, a retrospective study from the Paediatric Rheumatology European Society (PRES) Vaccinations Working Group reported good safety data of 234 patients with various rheumatic diseases receiving booster doses of MMR/MMRV while treated with various immunosuppressive treatments (40).

#### Other Vaccines

There are a few studies that have shown that the yellow fever vaccine was relatively safe in immunocompromised patients (solid organ transplant recipients, patients with immunosuppressive treatment like corticosteroids, mesalazine, MTX, and bDMARDs) and was not associated with infections by the attenuated vaccine strain (50–52). One case series has also assessed the immune response to a booster dose of yellow fever vaccine in 15 adults with various rheumatic diseases treated with MTX and anti-TNFα reported a similar antibody response to healthy controls and no adverse events, although there was a trend towards a lower immune response in patients, but due to the small sample size, no formal statistics could be performed (53).

#### What Do We Know and What Are the Controversies

Overall, these studies show that children treated with low-dose csDMARDs and GCs who received booster doses of MMR, VZV, or primary vaccination against VZV, had no severe adverse reactions and no cases of vaccine-derived viral infections or worsening of disease activity (35, 36, 43, 53). Therefore, even if larger studies are necessary, it appears that booster vaccinations with live-attenuated vaccines can be considered in patients with autoimmune disorders treated with various csDMARDs at low dose or GCs, or even some bDMARDs (54) However, more data are needed for these new treatments as they are more specific and, therefore, they could impact on a similar pathway needed for vaccine responses. An immunology work-up could also be done before vaccination with live-attenuated vaccines by looking at total lymphocyte count, IgG levels, vaccine antibody levels, including lymphocyte immunophenotyping looking at CD4 and CD8 counts and a T-cell stimulation test (4, 48).

Concerning immunogenicity, all these results show that live-attenuated vaccines induce a good immune response in the short term in children with various immune-mediated disorders on GCs, csDMARDs or bDMARDs (anti-TNF, anti-IL-1, anti-IL-6) (36, 38–40, 43, 45–48) as summarized in **Table 2**. However, a rapid loss of the antibodies can be expected in the longer term under immunosuppression, although persistence may be maintained with some csDMARDs. Results also suggest that the responses are lower in children on bDMARDs. These results are very important in the context of measle outbreaks occurring worldwide as immunosuppressed children not up to date with their vaccines are particularly at risk of infection. Booster doses may be needed, but it is difficult to establish common guidelines as to when booster should be given as the long-term effect may depend on the complexity of therapy.

#### Recommendations

The recommendations of the PRES concerning live-attenuated vaccines in children with rheumatic disease were published in 2011 (7) and updated in 2015 (54). According to PRES, live-attenuated vaccines against MMR and VZV can be given safely in children with rheumatic disease without immunosuppression according to national guidelines (7, 54). As soon as an immune-mediated disorder is suspected, screening for VZV and measles should be done systematically through infection and vaccine history and, if possible, confirmation by vaccine serology (4). If the surrogate marker is below the threshold considered protective, seronegative patients for VZV and measles should be vaccinated before the start of immunosuppressive/immunomodulatory therapy. Two vaccine doses, at least 1 month apart, should be administered and the last dose should be given ≥1 month before the start of immunosuppressive therapy (4, 7, 55, 56).

Under immunosuppression, it is recommended to first give a non-live vaccine (preferably following a novel antigen, such as hepatitis A) and assess the antibody response one month after vaccination, as well as to measure the number of CD4/CD8 cells. If the antibody response is good, including the T-cell numbers, a live-attenuated vaccine can be considered (4).

In general, live-attenuated vaccines are contraindicated under immunosuppressive therapy. However, as the replication potential of varicella vaccine is low and antivirals are available, varicella vaccine can be considered in any stable child under low-dose therapy with MTX, AZA, or 6-MP (4, 57), while MMR and yellow fever vaccinations can be considered in clinically stable patients during low-dosage GCs and MTX therapy ≤15 mg/m^2^/week (7, 54). According to other recommendations, booster vaccinations against VZV, MMR, and yellow fever, can also be considered in patients on low-dose csDMARDs (2, 4) (**Table 3**).

**Table 3:** Recommendations for live vaccines.

Vaccine	For whom	Dose and timing	Control of serology - short term- long term	Comments
Varicella	Seronegative for VZV**	two doses	Check serology after first dose if booster vaccination or after second dose if primary vaccination	- 4 weeks before starting immunosuppression - booster doses, may be considered under low-dose immunosuppression* if personal risk of exposure is high (5, 7, 13, 57)
MMR	Seronegative for measles**	two doses	Check serology after first dose if booster vaccination or after second dose if primary vaccination	- 4 weeks before starting immunosuppression. - Booster doses, may be considered under low-dose immunosuppression* if personal risk of exposure is high (5, 7)
Live typhoid vaccine	Only for travel in endemic regions, but use non-live vaccine			Contraindicated for immunosuppressed children, consider non-live polysaccharide vaccine (Typhim Vi®) (5)
BCG vaccine	Only for children returning definitively to endemic countries for tuberculosis			Contraindicated in immunosuppressed children
Yellow fever	Only for travel in endemic regions			- No data in children - Booster doses, may be considered under low-dose immunosuppression* if personal risk of exposure is high (5, 7)
Rotavirus	Follow local guidelines			Usually not applicable as should not be given after the age of 6 months (6)

*Low-dose immunosuppression as defined in **Table 1**.

**Correlate of protection as defined in **Table 5** (4, 10).

Live-attenuated vaccines should be avoided in children on high-dose immunosuppression (7, 54). Indeed, the replication of the live-attenuated vaccine may not be sufficiently controlled under strong immunosuppression and attenuated vaccines have the theoretical risk of a reversion to the virulent form, thereby inducing overt disease (49, 58). In the healthy population, this presentation is extremely rare, generally mild, and self-limited (23).

The interval after which a live vaccine can safely be given after interruption of the immunosuppressive drug is dependent on the pharmacokinetics and pharmacodynamics of the molecule. In principle, it is considered that five times the specific half-life of a drug corresponds to the time needed to clear the drug from the body, but the immunosuppressive effect can last longer. For anti-cytokine drugs, the immunosuppressive effects are expected to be of shorter duration than for drugs inhibiting cell division or cell function. Therefore, some guidelines recommend “5 × t1/2 elimination + immunosuppressive effect” (which is 2 weeks for anti-cytokines and 4 weeks for other drugs) (6). In general, it is recommended to wait for at least 4 weeks after discontinuation of high-dose GCs, at least 3 months after discontinuation of csDMARDs, and at least 3 months after discontinuation of a bDMARDs (56).

**Table 1** summarizes the effects of each immunosuppressive drug and the delay necessary before vaccination with a live-attenuated vaccine. These tables should be taken as indicative and not as strict guidelines according to expert consensus (2–5, 10, 56). Delays were calculated according to the half-lives of the drugs (usually five half-lives) and the expected duration of the immunosuppressive effect after interruption. The various delays can be followed before planning any live-attenuated vaccines in children on immunosuppressive treatments while considering the risk and benefit of vaccination in each situation.

It is also recommended to verify the vaccination status of the household and other close contacts and vaccinate them if indicated so as to minimize the risk for immunocompromised children through a “cocooning strategy” (56).

In addition, if there is no time to administer live-attenuated attenuated vaccines before starting immunosuppression, patients should be informed of their risk in the case of known exposure and advised to consult rapidly to receive prophylactic treatment antivirals/Igs (4).

Concerning other live-attenuated vaccines, they are usually contraindicated in patients on immunosuppression. The same recommendations should be followed for VZV and MMR vaccines. If travel is planned to an endemic country for yellow fever soon after the diagnosis, this vaccine should be administered before starting immunosuppression. In general, families should be discouraged from traveling to countries endemic for yellow fever, and other diseases for which only live-attenuated vaccines are available, these are contraindicated. Yellow fever vaccination can be given in clinically stable patients during low dosage MTX (4). If yellow fever vaccine has been already administered previously, an antibody measurement should be performed. Seropositivity indicates past immunity and enables travel to yellow fever endemic areas, regardless of the time that elapsed since immunization. As a precaution, oral typhoid vaccination (Vivotif^®^) and BCG vaccine should generally be avoided in all patients under immunosuppression (4).

## Vaccination With Non-Live Vaccines

Several studies have shown that non-live vaccines in children with autoimmune disorders treated with different immunosuppressive drugs do not worsen the disease or cause serious adverse events compared with healthy subjects as reviewed in detail in (7) and (54).

For children with rheumatic diseases, EULAR recommends adhering to national vaccination guidelines for diphtheria, Hib, hepatitis A and B, pertussis, pneumococci, poliomyelitis, meningococci, rabies, tetanus, and tick-borne encephalitis. Vaccination schedules differ among countries (31–33, 59). **Table 4** summarizes the recommendations for the various non-live vaccines.

**Table 4:** Recommendations for non-live vaccines (3–11, 31–33, 35–40, 42–49, 56, 57, 59).

Vaccine	For whom	Dose and Timing	Control of serology - short-term- long-term	Comments
**Influenza**	All	-one dose 1×/year during the influenza season -first year, two doses are recommended in patients <9 years	No (no correlate of protection)	Should also be administered to family members and close contacts.
**HBV**	Seronegative for anti-HBs (<10 UI/l)	See national vaccination schedule, usually: three doses at 0, 1, and 6 months, and two doses at 0 and 6 months for children 11–15 years	-Yes, 1 month after primary-immunization and then regularly if stayed under immunosuppression	Should be continuously >10 IU/l
**HAV**	-Seronegative for anti-HAV -frequent travellers	See national vaccination schedule, usually: two doses at 0 and 6 months	-Yes, 1 month after primary-immunization	
**HPV**	9–26 years	See national vaccination schedule, usually: three doses at 0, 1, and 6 months and two doses at 0 and 6 months for children 11–15 years	No (no correlate of protection)	
**PPV23**	Only recommended in some countries (North America)	one dose at least 8 weeks after PCV13		
**PCV13**	All, especially patients with low complement or functional asplenia (7)	See national vaccination schedule, usually one dose after general immunization in infancy	-Yes, if possible 1 month after vaccination -verify maintenance of antibodies regularly in children remaining on immunosuppression if possible	Give booster doses when below protective threshold
**Hib**	All, especially patients with low complement or functional asplenia (7)	one dose	-Yes, if possible 1 month after vaccination	
**Quadrivalent meningococcal conjugate vaccine**	All, especially patients with low complement or functional asplenia and those who will start eculizumab (7, 10, 59)	See national immunization guideline but usually, one dose - <5 years or between 11 and 15 years	No (no correlate of protection)	Doses to be repeated every 5 years if hyposplenism
**Serogroup B meningococcal vaccine**	All, especially patients with low complement or functional asplenia and those who will start eculizumab (7)	See national immunization guideline, but usually two or three doses depending on the vaccine	No (no correlate of protection)	Not licensed in all countries
**Diphtheria** **tetanus**	-Seronegative for tetanus	See national immunization guideline	-Yes, 1 month after primary-immunization and then regularly if stayed under immunosuppression	-Regardless of the age, pediatric formulation is recommended because of higher antigen concentration (6, 14) -tetanus Ab titer should be checked after vaccination if poor response is suspected (6) - depending on the age, use combined DTPa-IPV + -Hib/HBV
**Inactivated polio virus**	All, but particularly those who may travel to endemic countries	See national immunization guideline	No (no correlate of protection)	
**Pertussis**	Concerning pertussis: those in contact with small children	Schedule according to national plan	No (no correlate of protection)	
**Tick-borne Encephalitis**	Children living in or traveling to endemic regions (many countries in Western, Northern Europe; see WHO map)	three doses at 0, 2–4 weeks, and 6-12 months, then booster every 10 years	Yes, if possible 1 month after primary immunization	
**Typhoid fever**	In case of travel to endemic regions	Schedule according to national plan		Only this non-live vaccine is allowed in immunosuppressed children against typhoid fever
**Rabies**	In case of travel to endemic regions	Schedule according to national plan		

PCV-13, 13-valent pneumococcal conjugate vaccine; PPSV-23, 23-valent pneumococcal plain polysaccharide vaccine.

When a disease that potentially requires an immunosuppressive treatment is diagnosed, the vaccine status of the child should be verified. All non-live vaccines can be given without restriction, but they should be given 2 weeks before treatment starts to increase immunogenicity. When possible, the vaccine-specific antibody responses should be verified, especially following primary immunization and for children treated with high-dose immunosuppression and bDMARDs (7, 54, 55). **Table 5** shows whether a meaningful serological test is available. **Table 4** summarizes the general recommendations for each non-live vaccines.

**Table 5:** Correlate of protection for vaccine preventable diseases (4, 10).

Vaccine antigen	Correlates for protection	Test used for seroprotection	Susceptible	Short-term protection	Long-term protection	
	Units					
Diphtheria	IU/ml	ELISA	<100	100–999	≥1,000	
Tetanus	IU/ml	ELISA	<100	100–999	≥1,000	
Haemophilus influenzae type b (Hib)	UI/ml	ELISA	<0.15	0.15–0.99	≥1	
Hepatitis B	IU/ml	ELISA	<10	10–99	≥100	
Pneumococcal vaccination	mg/L	ELISA	<0.3	0.3–0.9	≥1	
Hepatitis A	IU/ml	ELISA	<20	≥20		
Measles	IU/ml	ELISA	<150	≥150		According to the ultrasensitive test
Varicella	IU/ml		<150	≥1500		According to the ultrasensitive test

### Influenza

#### Evidence for Immunogenicity

The response to influenza vaccine of children with rheumatic disease on immunosuppressive treatment has been widely studied, especially during the influenza A H1N1/2009 pandemic. It was observed in a case–control study of 95 patients with JIA compared to 91 healthy controls that the immune response was generally good, but sometimes associated with a reduced immune response in children with polyarticular JIA (60). In addition, another case–control study assessed the antibody response in 118 SLE patients and 102 healthy controls and reported that high disease activity was associated with a decrease in the antibody response to influenza A H1N1/2009 (61). Two other case–control studies of children with various rheumatic diseases (JIA, SLE, JDM) compared to healthy children reported a decreased immune response in those treated with high-dose GCs (62, 63) or combination treatment with GCs, MTX, and cyclosporin (63).

Therefore, the recommendation to vaccinate all children under the age of 9 years receiving the seasonal influenza vaccine for the first time with two doses at 1 month apart should perhaps be extended also to older immunosuppressed children. In a prospective case–control study, Aikawa et al. assessed the immunogenicity of two doses of the non-adjuvanted influenza A H1N1/2009 vaccine in children younger than 9 years with rheumatic disease compared to healthy controls and reported it to be safe and immunogenic in this patient population (64).

#### Recommendations

Seasonal influenza vaccination is recommended annually to all children with immune-mediated disorders treated or not with immunosuppressive drugs as influenza can be very severe and increase the risk of secondary bacterial infections (7). The first year, two doses are recommended in patients <9 years who have never been vaccinated against influenza or for whom the vaccination history is unknown (31–33, 59). The vaccination status of the household and other close contacts should be verified, and they should be encouraged to receive the current seasonal influenza vaccine.

### Hepatitis A

#### Evidence for Immunogenicity

Previous studies have reported a good immunogenicity of the hepatitis A vaccine (HAV) in children on immunosuppressive treatment, except in some conditions. Indeed, a case–control study assessing the antibody response to HAV in JIA and healthy controls reported a decrease of the antibody response in children with active systemic JIA on anti-TNFα (65). However, other case–control studies have reported high seroconversion rates following HAV vaccine in patients with IBD on infliximab (66), 6-MP, or AZA (67) compared to healthy controls.

#### Recommendations

HAV should be offered to seronegative children with autoimmune disorders who travel frequently to endemic countries. The schedule should follow national guidelines (31–33, 59). A control of the response to HAV is recommended in immunosuppressed children by serology (4). If short-term protection is necessary, a serology can be performed 1 month after the first dose and, if necessary, a second dose can be administered at a short interval. For long-term protection, a serology should be performed 1 month after the last dose (6 months after the first dose) and, if necessary, further vaccine doses should be administered (4).

### Hepatitis B

#### Evidence for Immunogenicity

Several case–control studies in children suffering from various immune-mediated disorders (autoimmune hepatitis, IBD, and JIA) have observed a reduced immune response following HBV vaccine in children with immune-mediated disorders compared to healthy children and a decreased long-term antibody persistence, particularly in those treated with GCs, AZA, and anti-TNFα (68–71). In another case–control study, 14 children with IBD non-responders to three doses of hepatitis B vaccine (20 mcg) received a booster dose of hepatitis B vaccine. After this additional dose, 7/14 (50%) seroconverted. Overall, seroprotection was 85% after a full vaccination scheme and a booster dose (72), even if an adjuvant was used (aluminium), suggesting that this may not be sufficient.

#### Recommendations

Hepatitis B vaccination is recommended for children with autoimmune disorders because of potential severe disease during immunosuppression. All children should be screened by serology soon after the diagnosis. Hepatitis B should be administered to children seronegative (without anti-HBs antibodies) according to national guidelines (31–33, 59), which is three doses at 0, 1 and 6 months for most countries (and in some countries two doses at 0 and 6 months for children 11–15 years). If protection is needed more rapidly, the accelerated scheme (1, 7, 21 days, 6–12 months) is indicated, *e.g.* patients who need to start rapid immunosuppression. In the case that the family travels extensively and no natural immunity against hepatitis A has been acquired yet, the combined hepatitis A and B vaccines (Twinrix^®^) should be chosen, as it is known to be more immunogenic than the monovalent HBV. It is recommended to verify the antibody titer one month after the 3rd vaccination (scheme 0, 1, and 6 months) and after the 4th dose if the scheme is 0, 7, 21 days and 6–12 months. Levels of anti-HBs >100 mIU/ml should be achieved. If necessary, booster doses should be administered. There are no data on the maximal doses to be given in the case of an absence of response, but usually up to six doses are given. In addition, Twinrix^®^ can be given in immunosuppressed children in the case of an absence of response (usually three doses at 0, 1, and 2 months) according to a recent study (73).

Maintenance of HBs antibody should be monitored on a regular basis in immunosuppressed children. A booster dose of hepatitis B vaccine should be given if anti-HBs fall below 10 IU/l (4, 6).

### HPV

#### Evidence for Immunogenicity

Heijstek et al. assessed the immunogenicity and safety of the bivalent HPV in young females with JIA, SLE, and JDM in a case–control study and reported lower antibody and memory B cell concentrations in patients compared to healthy controls, although not statistically significant. There was no significant effect of the various immunosuppressive treatments (MTX and anti-TNFα) on the immune response to HPV vaccine. However, it has been reported in two case–control studies of patients with JIA or JDM that the antibody concentrations tended to be lower in patients than in healthy controls. This was even observed in adolescent girls who were not receiving any immunosuppression, due to an unclear mechanism that remains to be elucidated (74, 75), despite the fact that the vaccine was adjuvanted with aluminium. Similarly, an adult case–control study reported a reduced immunogenicity of the quadrivalent HPV vaccine in adult patients with SLE compared to healthy controls (76).

#### Recommendations

HPV vaccination is recommended for young adults aged 11–26 years with autoimmune disorders according to the national vaccine schedule (31–33, 59). The immunogenicity results of previous studies suggest that assessing the vaccine response and antibody persistence following HPV vaccination in this population may be useful, although there is still no recognized seroprotection cut-off for this age group.

### Pneumococcal Vaccines

#### Evidence for Immunogenicity

In a case–control study of JIA children and healthy controls, Farmaki et al. observed that following the pneumococcal protein 7-valent conjugate vaccine (PCV7), JIA-children had a normal antibody response when treated with MTX or cyclosporine, either with or without GCs, but a lower antibody response if treated with anti-TNFα (77). Other studies have reported severe local and systemic reactions following the plain polysaccharide pneumococcal vaccine in children and young adults with an autoinflammatory disease, such as cryopyrin-associated-periodic syndromes (78, 79), but probably due to the disease itself and not due to the therapy (inflammasome trigger). Therefore, for this population of children, the PCV may be better.

#### Recommendations

Both the PCV13 and PPV23 pneumococcal vaccines are still recommended in many countries, such as the USA, Canada, Cyprus, Greece, France, and Spain (31–33, 59). For example, the US Advisory Committee on Immunization Practices recommends the following vaccination plan for children with chronic diseases: four doses of PCV13 at 2, 4, 6 and 12–15 months of age, followed by two additional doses of PPV23 at 5 years’ interval between the ages of 2 and 18 years (32). The rationale being that the PPV23 adds protection against a larger number of serotypes than the PCV13. However, because of its T-independent characteristic, it only induces short-term immunity and weaker immune responses than the PCV13, which is a T-dependent antigen (57). For this reason, only PCV13 is recommended in Switzerland. In general, conjugate pneumococcal vaccine should be preferred over polysaccharide vaccine as conjugate vaccines produce higher affinity antibody responses, longer lasting immune responses, as well as the production of memory B cells. Booster vaccinations after conjugate vaccines permit an amplification of the pool of memory B and T cells. In contrast, booster vaccination with plain polysaccharide vaccines may deplete the pool of memory B cells due to a lack of induction of memory cells (80). The fact that some countries still include the polysaccharide vaccine in their recommendations depends on the pneumococcal serotype distribution circulating in the country.

Vaccination against pneumococcal disease is recommended for all children with autoimmune disorders according to national immunization guidelines (31–33, 59). Ideally, the vaccination should be administered prior to the start of immunosuppressive therapy. If immunosuppressive therapy has already been started, the vaccination should be administered at a time point when the level of immunosuppression is lowest. Whether and when booster vaccination may be needed following PCV13 priming remains to be defined. Immunogenicity may be reduced under some immunosuppressive treatments and, if possible, verification of the immune response should be performed 1 month after vaccination and regularly in children remaining under immunosuppression (4).

### Meningococcal Vaccines

#### Evidence for Immunogenicity

Stoof et al. conducted a retrospective cohort study of the kinetics of specific antibody responses following the meningococcal serogroup C-conjugate vaccine over time in children with JIA. They observed a similar antibody response and waning of meningococcus-specific IgG titers over time in patients and healthy controls. However, the loss of antibodies was more rapid in patients on bDMARDs than on csDMARDs (81).

#### Recommendations

Monovalent (capsular groups A and C) or quadrivalent (MenACWY) meningococcal conjugate vaccine, as well as the vaccine against serogroup B (MenB), is recommended in several European countries and in the USA and Canada, depending on the endemicity of the various meningococcal serogroups in the different locations worldwide (31–33, 59). Patients with acquired complement deficiency, such as patients receiving the monoclonal antibody eculizumab, and other children who are receiving an immunosuppressive treatment are also at risk of hyposplenism and should be up to date with their meningococcal immunization (10).

### Tetanus-Diphtheria-Acellular Pertussis-Polio Vaccines

#### Evidence for Immunogenicity

In general, the antibody response to tetanus–diphtheria vaccination is similar in patients with immune-mediated disorders and healthy controls. However, case–control studies assessing measles and tetanus antibodies in children with SLE observed that the antibody titers tended to decrease more rapidly in patients treated with immunosuppressive drugs (37, 38). Another retrospective, cross-sectional study in children with various rheumatic diseases and healthy controls also reported a decrease in antibody in children with rheumatic disease (37, 38).

#### Recommendations

Tetanus, diphtheria, acellular pertussis, and poliomyelitis vaccinations are recommended for all children with immune-mediated disorders, according to the national immunization guidelines specific to the country (31–33, 59). The timing and number of doses depend on the number of previous doses received and the interval since their last dose of vaccination. In young adults, after primary vaccination, booster doses of diphtheria/tetanus vaccine should probably be given more frequently than in healthy persons, *i.e.* every 10 years (4).

### Haemophilus Influenzae Type B

#### Recommendations

Hib vaccination should be administered according to national immunization guidelines (31–33, 59). Based on the current epidemiology, Hib immunization is not recommended after the age of 5 years, even in immunosuppressed patients, except in the Czech Republic, Greece, the USA, and Canada (31–33, 59).

### Other Vaccines: Rabies, Japanese Encephalitis, Parenteral Typhoid Vaccines, Tick-Borne Encephalitis

#### Evidence for Immunogenicity

In the literature, no data were found on the safety and immunogenicity of the inactivated vaccinations against rabies, Japanese encephalitis, typhoid fever, or tick-borne encephalitis in children with autoimmune diseases.

#### Recommendations

Vaccinations against rabies, Japanese encephalitis, or typhoid fever are indicated for specific risk situations according to national immunization guidelines before traveling to endemic areas (31–33, 59). The indications should be discussed individually with specialists before planning international travel.

A vaccination against tick-borne encephalitis is recommended for children with an increased risk of exposure according to the national immunization guidelines for each country (32, 59). The usual course of vaccination should be followed (three dose-scheme with a booster dose every 10 years). In immunosuppressed patients, a serology should be performed 1 month after the last dose.

### Particular Situations

#### Children Treated With Intravenous Immunoglobulin

In the case of treatment with IVIg, the immune response to live-attenuated vaccines may be reduced if the vaccine is administered immediately before or 1–4 weeks (or more) after IVIg. Live vaccines should be given either 2 weeks before or should be delayed for 3–11 months after IVIg, depending on the dose. In the case of treatment with IVIG within 14 days of a live vaccine, the vaccine should be verified after 3–11 months after IVIg treatment, and the vaccine re-administered if necessary.

#### Children Treated With B-Cell Depleting Drugs

There are no data on the immune response post-vaccination in children treated with anti-CD20. In a prospective controlled study, Oren et al. assessed the humoral immune response to the seasonal influenza vaccine in three groups, *i.e.* 29 adults with rheumatoid arthritis, 14 rheumatoid arthritis adults treated with rituximab in the previous 18 months, and 21 healthy adults. They observed that patients treated with rituximab responded less well compared to the two other groups but still developed a partial immune response to the seasonal influenza vaccine (82). Nagel et al. showed in a prospective controlled study that belimumab given in addition to csDMARDs did not decrease the antibody response to PCV13 in SLE patients (83). In a phase 4, open-label study among patients randomized to receive the PPV23 either 4 weeks prior to belimumab or 24 weeks after starting 4-weekly belimumab treatment, Chatham et al. observed that both groups responded similarly to the PPV23 (41). Other groups have shown that there is very little antibody induced, but T cells may be preserved (unpublished).

During 6–9 months following treatment with anti-CD20 monoclonal antibodies or anti-BLys, immune responses to vaccination are severely impaired as many antibody-producing plasma cells are short-lived and require replacement from CD20+ precursors. In addition, the number of memory B cells in the bone marrow is also reduced (84), and B cells returning from the bone marrow to the peripheral blood have an immature phenotype (CD27−IgD−) or naïve (CD27−IgD+) rather than memory B cells. The development of new memory B cells appears to be delayed for many years. However, it appears that long-lived plasma cells may not be affected by B cell depleting drugs. Therefore, it is recommended to administer primary immunization before anti-CD20-depleting antibodies. Secondary immunization can be administered 6 months after these treatments for non-live vaccines, but only after 12 months for live-attenuated vaccines (5, 6, 29). Prolonged hypogammaglobulinemia and B-cell depletion have been reported following rituximab. Since there are recommendations to document prior to therapy and then monitor Ig and B-cell levels during therapy, it may be reasonable to ensure that these levels have normalized prior to any immunizations.

Although the immune response is expected to be diminished in individuals under B cell-depleting drugs, the seasonal influenza vaccine is still recommended, as we expect to induce a T-cell immune response (4).

#### Infants Born to Mothers Who Received Immunosuppressive Treatment During Pregnancy

As some immunosuppressive drugs pass the placental barrier, they can be found in new-borns for 6–8 months, especially if they are taken at the end of pregnancy by mothers. These drugs can affect the development of the immune system of the new-borns and also affect the response to vaccination. For example, a case of fatal “BCGitis” has been reported in a 3-month-old infant whose mother had been treated with infliximab during pregnancy (85). Drugs such as MTX, MMF, leflunomide, and cyclophosphamide are teratogenous and contraindicated during pregnancy (86). Other medications such as antimalarials, sulfasalazine, AZT, cyclosporine, tacrolimus, and colchicine are not immunosuppressive and can be administered during pregnancy (86). COX2 selective non-steroidal anti-inflammatory drugs (NSAIDs) and corticosteroids can be given until 28 gestational weeks (86). In severe refractory maternal disease during pregnancy, pulses of methylprednisolone and IVIg can also be given until the end of pregnancy if necessary. It should be noted that biological monoclonal antibodies are transferred through the placenta, like other Igs, from week 13 until the end of pregnancy, with a peak during the last 4 weeks of pregnancy, resulting in a blood level 120–130% higher than the blood levels of the mother. Then, it appears that the half-life of the biological molecules is prolonged in new-borns (infliximab can be measured for up to 6–12 months in babies, adalimumab for 3–6 months). Concerning anti-TNFα, they can be given during the two first trimesters and it seems that etanercept and certolizumab can also be given until the end of pregnancy due to a low rate of transplacental passage. Other bDMARDs should not be used during pregnancy (86).

EULAR recommends vaccinating infants according to the normal schedule if biological agents have been discontinued before week 22 of gestation. However, if immunosuppressive treatment is continued past 22 weeks in the mother, live-attenuated vaccines (including BCG, rotavirus, oral polio, MMR and VZV) should be given after the age of 6 months. It is also possible to measure the metabolite levels in the blood of the infant. In contrast, inactivated vaccines can be given according to the normal schedule (86).

Most csDMARDs, bDMARDs, and tsDMARDs are contraindicated during breastfeeding, except for antimalarials, sulfasalazine, AZT, cyclosporine, tacrolimus, colchicine, prednisone, Ig, and anti-TNF because of a low transfer to breast milk. Therefore, children who are only exposed to those immunosuppressive drugs during breastfeeding can be vaccinated normally (86).

#### Proposed Practical Approach Concerning the Vaccination of Children With Immune-Mediated Disorders Who Should Start Immunosuppression or Already Under Immunosuppressive Treatment

##### In A Child Newly Diagnosed With an Immune-Mediated Disorder

- Check the immune status of the child through natural infection and vaccine history and serology.
 Administer missing vaccines according to the age and condition if possible and prior to initiation of immunosuppressive treatment.
- For non-live vaccine, 2 weeks are generally required for the development of the immune response following primary immunization and around 1 week following booster immunization.
 Verify when possible the vaccine response 1 month later and if vaccine antibodies remain below protective threshold as defined in **Table 5**, administer additional doses of vaccine.
- For live-attenuated vaccine, if time is sufficient (at least 4 weeks), administer one or two doses of the live-attenuated vaccine at 4 weeks interval. Verify the response after the first or second dose.
- However, in a case of good epidemiological situation, it is better not to vaccinate during an active disease, and to wait that the disease is stable on therapy to vaccinate, at least with non-live vaccines.

##### In Children Already Under Immunosuppression

- Define the effect of the underlying disease and/or immunosuppressive treatment on the immune response to vaccination.
- Using **Table 1**, determine how the immunosuppressive treatment will affect the cellular and humoral immune response to vaccines and whether any precautions are required before administering non-live or live-attenuated vaccines.
- For non-live vaccine, all vaccines are allowed; however, the immune response may be diminished with high-dose immunosuppression. Therefore, it is recommended to check the antibody response 1 month after vaccination when possible.

##### If a Live-Attenuated Vaccine Is Required Despite Immunosuppression

- Live-attenuated vaccines are usually contraindicated during immunosuppressive treatment. They can be considered in some circumstances if the personal risk of exposure to a given disease is high.
 If the child has already received in the past the vaccine antigen for which he has no longer protective antibody levels, revaccination can be considered under certain conditions (low-dose immunosuppression.
- If primary immunization with a live-attenuated vaccine is needed, depending on the planned duration of the treatment and risk of exposure to the pathogen, it may be possible to interrupt temporarily the immunosuppressive treatment. In this case, follow **Table 1** showing the minimal time interval between interruption of a certain immunosuppressive treatment and the administration of live-attenuated vaccines. However, the risk of exacerbation of the autoimmune disorders should also be considered.

##### When the Vaccine Antibodies Should Be Verified

- Under immunosuppression, the antibody response to primary immunization should be verified at 1 month post-vaccination when possible as the immune response may be decreased depending on the treatment, and additional doses may be necessary.
- Secondary immunization is expected to give rise to protective antibody levels, although at lower levels and for a shorter duration than in healthy children. Therefore, it is advisable to regularly assess vaccine antibody in a short period after vaccination (few month) and in the long-term (years) because children treated with immunosuppressive therapy might need booster doses more often than healthy children might. The timing of revaccination should be decided according to vaccine serology. This is particularly important for those pathogens that present a significant risk for community acquisition because of poor vaccine uptake (measles, varicella) and/or pathogens that present a significant lifelong risk and for which regular boosters are recommended (tetanus, pneumococci, hepatitis B).

##### Patients Treated With IVIG

- These treatments are not considered as immunosuppressive. However, they affect the immune responses to vaccinations.
 First, vaccine serology is not reliable.
- The responses to live-attenuated vaccines are altered as the Igs will inhibit the replication of the live-attenuated virus. Therefore, a delay of 3–11 months is recommended between the end of the IVIG and the administration of live-attenuated vaccine, depending on the IVIG dose. It is also recommended to verify that vaccine antibodies (passively transferred through the IVIG) have disappeared before administering live-attenuated vaccines.
 There is no delay necessary between non-live vaccines and IVIG perfusion.

## Discussion

Overall, there are many studies concerning the vaccination of children with autoimmune disorders; however, there are still many open research questions (**Table 6**).

**Table 6:** Open research questions.

How often should we assess the vaccine serology in patients on immunosuppressive treatment for the various vaccine antigens: pneumococci, tetanus, measles, varicella, hepatitis A and B?
How often vaccine booster doses should be given in children on immunosuppressive treatments? Especially for pneumococcal vaccines
What is the correlate of protection that we should aim in children on immunosuppressive treatment (especially for pneumococci, measles, varicella) ?
What are the factors affecting the antibody response in the short-term and the speed of decline of antibodies in the longer term (type and dose of immunosuppressive treatment, previous vaccinations, time since last vaccines, age, activity of the disease) ?
Should we give supplementary doses of vaccines in children on immunosuppressive treatment, for example against influenza or hepatitis B, or other vaccines?
How antibody levels correlate with long-term protection? Do we have better correlate of protection?
Can we develop other immunological tests (such as the measurement of specific memory B and T cells) to assess long-term protection?
Should we develop new vaccine strategies (such as the use of new adjuvants or the use of DNA vaccines) to increase vaccine protection among these children?
Can we develop algorithm to better define under which treatment we can safely administer live attenuated vaccines? Can we perform an immunology workup which can predict that it is safe to give a live attenuated vaccine?

All published studies are very reassuring from a safety point of view, and most vaccines appear to be safe in children with autoimmune disorders on immunosuppressive treatment. They do not frequently cause serious adverse events and do not increase disease activity. However, there are only a few studies that have assessed vaccination with live-attenuated vaccines in this patient population.

It appears to be safe to vaccinate children treated with low dose csDMARDs and GCs, including booster doses of MMR and VZV or primary vaccination against VZV, as there has been no report of severe adverse reactions or cases of vaccine-derived viral infections or worsening of the disease activity under these conditions. However, larger studies are necessary to define the exact conditions under which live-attenuated vaccines can be given in children on high-dose DMARDs, bDMARDs. Indeed, live-attenuated vaccines can be considered on a case-by-case basis for children with autoimmune disorders treated with higher immunosuppression. For these patients, it is important to have a systematic approach to assess vaccine status and to plan the vaccinations at a specific time of the disease.

Concerning immunogenicity, most immunosuppressive treatments at low dose induce a normal antibody response in the short term. However, immunogenicity of some vaccines under higher immunosuppression is less clear. Although all non-live vaccines can be given even under high immunosuppression, it is not always very clear how the child will respond to the vaccination. Therefore, when possible, it is important to assess the antibody response 1 month after vaccination, as it might be necessary to give a supplementary dose of vaccine for some children.

Most studies have analyzed the short-term responses post-vaccination in children with immune-mediated disorders. However, long-term protection depends on persisting antibody levels above the threshold of protection until we know if the immunological memory can act rapidly enough to induce protective antibody levels in case of infection. Of note, this threshold of protection has been only established in healthy children and may be different in immunocompromised children. Therefore, a correlate of protection needs to be defined for this specific population to ensure that long-term protection is maintained. Therefore, it is very important to verify that children treated continuously with immunosuppressive treatment or suffering from various immune-mediated disorders that can affect their response to vaccination maintain protective antibody in the long-term. Indeed, it has been observed that the specific antibodies post-vaccination wane more rapidly in immunocompromised children than in healthy children. The speed of decline of the specific antibodies post-immunization in this population of children with immune-mediated disorders may depend on various parameters, such as the type and dose of immunosuppressive treatment, previous vaccinations, time since last vaccines, age, and the activity of the disease, but more studies are needed to define the exact factors that affect the rapidity of this decline. It is important to recommend how frequent the vaccine serology should be assessed in this population and how often vaccine booster doses should be given. For the moment, antibody persistence should be assessed more systematically in all children on immunosuppressive treatments, especially those on bDMARDs and against diseases for which the risk of exposure is continuous, such as pneumococci, influenza, tetanus, hepatitis B, VZV, and measles. There is also a need to develop laboratory tests, which are more widely available to help in monitoring long-term immunity to all vaccine-preventable diseases in high-risk children.

Assessment of immunity is largely restricted to antibody responses because of the difficulty measuring B- and T- cell-specific responses outside of specialized laboratories. However, long-term protection is more complex than just measuring the level of neutralizing antibodies. The quality of antibody and B cells (function, repertoire) is also important. There are only few data on recall responses in immunocompromised children and no data on B cell memory functions. It is well known that antibody levels can be under the protective threshold, although the individual may still be protected by the memory immunity, which can be re-activated very rapidly for some antigens, at least in healthy individuals. Studying the antibody and cellular immune responses in the short- and longer-term post-vaccination in this vulnerable population is crucial for the improved development of vaccine strategies (such as the use of new adjuvants or the use of DNA vaccines) to increase vaccine protection among these children.

## Conclusion

Vaccination of immunocompromised children is safe with non-live vaccines and in specific situations also with live-attenuated vaccines, and should be a priority and a concern of every doctor in charge of these patients. As soon as an autoimmune disorder is suspected, vaccine status should be verified and all missing vaccines administered if time is sufficient. During immunosuppression, immune responses may be decreased, especially in children treated with high-dose csDMARDs or bDMARDs. In addition, antibody titers decrease more rapidly in the long term than in healthy children. Therefore, the vaccine response should be assessed not only at one month post-immunization when possible, but also on a regular basis for some antigens like tetanus, pneumococci, and hepatitis B, in order to administer booster doses when necessary. Of note, it is important to determine who has the responsibility for these assessments among the health care providers of these children. Specialists are often the primary care providers of those complex patients and should be more pro-active in verifying the vaccine status of their patients and making recommendations as to when to administer particular vaccines.

Even if larger studies are needed, live-attenuated vaccines appear to be safe under low-dose immunosuppression or after temporarily interrupting immunosuppressive treatment. It would be helpful to have studies that test a specific protocol to assess immune competence before vaccinating with live-attenuated vaccine, such as giving first the hepatitis A vaccine, assess the antibody titer after one month, and vaccinate with live-attenuated vaccines if the response to hepatitis A is good and if the total lymphocyte count is good enough. Indeed, clear guidelines are thus needed in order to define in which situations live-attenuated vaccines can be used.

Finally, new vaccine technologies are exploding in the context of the SARS-CoV-2 pandemic, based mostly on gene analysis to indicate targets and then combining this information with new ways to target and “trick” the immune system into responding appropriately. Similarly, a better understanding of the pathology of immune-mediated disorders has led to the development of more targeted approaches. It is hoped that the current pandemic will also trigger the development of an array of vaccines to be used in different populations for the same pathogen, with special vaccines for immunocompromised children.

## Author Contributions

The author confirms being the sole contributor of this work and has approved it for publication.

## Conflict of Interest

The author declares that the research was conducted in the absence of any commercial or financial relationships that could be construed as a potential conflict of interest.

## Publisher’s Note

All claims expressed in this article are solely those of the authors and do not necessarily represent those of their affiliated organizations, or those of the publisher, the editors and the reviewers. Any product that may be evaluated in this article, or claim that may be made by its manufacturer, is not guaranteed or endorsed by the publisher.
